# Operative Techniques for Cervical Radiculopathy and Myelopathy

**DOI:** 10.1155/2012/794087

**Published:** 2011-08-14

**Authors:** R. G. Kavanagh, J. S. Butler, J. M. O'Byrne, A. R. Poynton

**Affiliations:** Department of Orthopaedic Surgery, Cappagh National Orthopaedic Hospital, Finglas, Dublin 11, Ireland

## Abstract

Cervical spondylosis is a common problem encountered in modern orthopaedic practice. It is associated with significant patient morbidity related to the consequent radiculopathic and myelopathic symptoms. Operative intervention for this condition is generally indicated if conservative measures fail; however there are some circumstances in which urgent surgical intervention is necessary. Planning any surgical intervention must take into account a number of variables including, but not limited to, the nature, location and extent of the pathology, a history of previous operative interventions, and patient co-morbidities. There are many different surgical options and a multitude of different procedures have been described using both the anterior and posterior approaches to the cervical spine. The use of autograft to achieve cervical fusion is still the gold standard with allograft showing similar results; however fusion techniques are constantly evolving with novel synthetic bone graft substitutes now widely available.

## 1. Introduction

Cervical spondylosis is a common problem that is increasing in incidence in our aging population. Presentation is usually with neck pain, cervical radiculopathy, cervical myelopathy, or a combination of these. 

The pathogenesis of cervical spondylosis is age-related degeneration with loss of disc height and posterior or posterolateral disc herniation. Degenerative changes also result in bulging of the ligamentum flavum which can impinge on the spinal cord posteriorly, osteophyte formation, and ossification of the posterior longitudinal ligament which can compress the spinal cord anteriorly [1].

Cervical radiculopathy has an incidence of 83.2 per 100,000 [2] with a prevalence of 3.5 per 1,000 population [3]. As cervical myelopathy is a rarer condition, there is little reliable epidemiological data. 

Radiculopathy is caused by nerve root compression and presents with dermatomal and myotomal dysfunction in the upper limbs with general lower motor neuron signs of weakness, wasting, flaccid paralysis, and hyporeflexia. Specific tests used in the setting of cervical radiculopathy include Spurling's test and manual cervical distraction; both of which may help to distinguish neurological pathology from other causes of a similar clinical picture. Myelopathy can present with a variety of symptoms: general upper motor neuron signs of weakness, spasticity, and hyperreflexia in both upper and lower limbs with Hoffmann's and Babinski's signs in the upper and lower limbs, respectively, as well as bowel and bladder dysfunction, clonus, myelopathic gait, sensory disturbances, and rarely a history of Lhermitte's sign. On examining the patient one may also elicit a positive inverted radial reflex and the finger escape sign.

## 2. Indications for Surgery

There are no strict guidelines on the indications for surgery in cervical spondylosis. The decision to proceed with surgery is taken after detailed consultation, physical examination, and imaging and is based on a number of variables including the severity of symptoms, duration of symptoms, progression of symptoms, radiological changes, and the patient's fitness for surgery. The failure of conservative management strategies, such as physiotherapy, analgesia, nonsteroidal anti-inflammatory drugs, and epidural injections, is another indication for surgical intervention.

It is generally accepted that in the setting of myelopathy, a shorter duration of symptoms before surgical intervention is associated with better neurological recovery, and this has been borne out in a number of studies [4, 5]. Indications for urgent surgery include new-onset gait disturbances, bowel/bladder dysfunction, and rapid progression of disease.

## 3. Planning Surgery

Both the anterior and posterior approaches can be utilised in accessing the cervical spine. The approach is dictated by a number of different variables including the location of pathology and type of procedure to be undertaken, previous surgeries to the area, extent of disease (single or multilevel), preoperative neck pain, the presence of congenital stenosis, sagittal alignment of cervical spine, and patient comobidities [6]. 

The exact nature and location of the pathology plays an important role in deciding which approach to take to the cervical spine. Posterolateral herniation of the intervertebral discs lends itself to either an anterior or posterior approach [7]; however central posterior herniation is better accessed through the anterior approach with fewer postoperative complications [8]. Whatever approach is taken, it is important to minimise working around the spinal cord so as to minimise the risk of spinal cord injury.

Previous surgery using the anterior approach can make subsequent surgeries more difficult due to the presence of scar tissue which increases the risk of damage to structures in the anterior neck. Contralateral anterior approach is possible, but preoperative laryngoscopy should be performed beforehand to outrule the presence of subclinical vocal cord paralysis due to previous injury to the recurrent laryngeal nerve on that side. Repeated surgeries to the posterior neck increase the risk of postoperative axial pain and paraspinal muscle dysfunction. [9–11].

The extent of the disease and the number of levels to be operated on are other important considerations in the planning of any surgery to the cervical spine. For one- or two-level disease that is accessible from the anterior, it is that approach that is generally favoured by surgeons. Patients with pathology at multiple levels should be considered for posterior approach as studies have shown similar neurological outcomes compared to anterior approaches but decreased operating time and complications in patients undergoing posterior surgery for multilevel pathology [12–15]. 

The presence of preoperative neck pain is a relative contraindication to posterior approach given the increased incidence and possible worsening of axial neck pain in patients undergoing a posterior approach [12, 16]. Therefore in patients with a significant degree of neck pain preoperatively an anterior approach is indicated if the pathology can be accessed through that approach. Studies have also shown that if a posterior approach is taken the incidence of postoperative axial neck pain is reduced with reduced number of laminoplasty levels [17, 18].

A normal mid sagittal cervical spinal canal diameter is 17-18 mm with congenital cervical canal stenosis defined as an AP diameter of <13 mm. Congenital stenosis increases the risk of developing cervical myelopathy or radiculopathy later in life due to even mild spondylosis and therefore is an important consideration in those presenting for evaluation [19–21]. Patients with developmental canal stenosis are often not suitable for anterior cervical discectomy and fusion (ACDF) due to high rates of postoperative clinical deterioration in this group after ACDF [22]; however laminoplasty has been shown to improve outcomes in patients with cervical spondylosis and concomitant congenital canal stenosis [23]. 

Sagittal alignment of the cervical spine is another consideration with mounting evidence to suggest that better outcomes are achieved by using the anterior approach compared to the posterior approach in patients with a kyphotic cervical spine [24, 25].

Spinal cord signal changes on preoperative MRI are another factor that affects postoperative outcomes with many studies showing poorer outcomes in patients with preoperative intramedullary hypointense signal changes on T1-weighted MRI [26–28].

## 4. Operative Techniques

The main procedures that are performed through an anterior approach are anterior cervical discectomy and corpectomy, and those carried out through a posterior approach are laminoplasty, laminectomy, and posterior cervical discectomy.

### 4.1. Anterior Cervical Discectomy

Anterior cervical discectomy is performed with the patient in the supine position with neck in slight extension. A transverse incision is made in the anterolateral aspect of the neck with dissection through the natural fascial planes of the neck between the carotid sheath laterally and the trachea and oesophagus medially. This allows good access to the cervical intervertebral spaces. Through this approach the surgeon can achieve decompression by discectomy or corpectomy. Additional procedures that can be carried out include removal of ossified posterior longitudinal ligament, osteophyte removal, and foraminotomy. ACDF is the procedure of choice for single-level disc disease and is also commonly performed for two-level disease. Studies have shown that for adjacent two-level disc disease, ACDF is superior to single-level corpectomy in terms of operating time and blood loss, but the two procedures have similar neurological outcomes [29, 30]. Postoperative dysphagia is a common complication after anterior surgery and has been reported to persist at one-year followup in 13–21% of cases and is higher in females and after multilevel surgery [31].

### 4.2. Corpectomy

Corpectomy is the removal of a central portion of the body of a vertebra. It can be used for the treatment of multilevel disease that is amenable to the anterior approach. It is used as an alternative if multiple discectomies and fusions are required and in cases where a large access area is required to completely decompress the spinal cord [6]. Symptoms due to short segment ossification of the posterior longitudinal ligament can be treated with corpectomy also [32]. Following corpectomy there are a number of options for filling the defect: iliac crest autograft is still the method of choice for one- and two-level corpectomy, but following multilevel corpectomy the use of a fibular strut allograft or metallic cages are reconstructive options. As for ACDF these can all be supplemented with anterior plating to provide extra stability and increase fusion rates. Newer techniques that have shown promising results include skip corpectomy and combined corpectomy and adjacent level discectomy [33, 34].

### 4.3. Laminoplasty

A laminoplasty is performed with the patient in the prone position through a posterior midline incision. The paraspinal muscles are stripped from the vertebrae before laminoplasty is performed. A laminoplasty can be performed using a single-door or double-door technique, and this can be supplemented by bone graft or instrumentation to keep the “door” open. For a single-door laminoplasty the junction of the lateral masses and the laminae is divided completely on one side while the other is divided to the anterior cortex; this side is then used as a hinge to rotate the other side open. For a double-door laminoplasty a midline osteotomy through the spinous processes and laminae is performed, and bilateral hinges are created by a similar technique as for the single-door laminoplasty. The laminae are then opened in the midline using the lateral hinges as axes of rotation. As with the single-door laminoplasty the double-door laminoplasty can be supplemented by instrumentation to maintain the decompression. Overall, results of single- and double-door laminoplasty show similar neurological outcomes [35] with canal expansion slightly more in the single-door group [36]; however, there may be certain subsets of patients that would benefit more from one procedure over the other (e.g., patients with myelopathy and bilateral radiculopathy will benefit more from double-door laminoplasty) [36]. Standard laminoplasty is performed from C3–C7; however, some studies suggest that decreasing the number of levels and surgical techniques to preserve the paraspinal musculature will improve postoperative axial pain [17, 37].

### 4.4. Laminectomy

Laminectomy is an alternative for posterior approaches to the cervical spine. This involves decompression by removal of the spinous processes and laminae at the levels to be decompressed. Laminectomy is often carried out with concomitant fusion to increase the stability of the cervical spine. This can also be supplemented with instrumentation to provide immediate stability and increase fusion rates. Laminectomy has been shown to provide excellent neurological and functional outcomes in multilevel cervical myelopathy [38, 39]; however, compared to laminoplasty it does show increased operating time and increased complications rates [40, 41].

### 4.5. Posterior Cervical Discectomy

Cervical discectomy is most commonly performed through an anterior approach; however, there are some circumstances where a posterior discectomy is performed. This includes where the anterior approach would be associated with an unacceptable complication risk and where a posterolateral herniated disc is easily accessible by a posterior approach [7]. The advantage of the posterior approach is that potential complications associated with the anterior approach are avoided, and fusion is not necessary so full cervical range of motion is maintained [42]. One disadvantage of this approach is the increased risk of nerve root and spinal cord injury.

## 5. Fusion Techniques

Fusion is performed with the placement of graft between the fusion surfaces followed by a period of immobilisation to allow the fusion to occur. Bone graft can be autograft, allograft, or synthetic bone graft substitutes. Autograft is still the gold standard with its long established safety and efficacy. Bone is usually harvested from the iliac crest which introduces the risk of complications relating to nerve or arterial injury, hematoma, infection, and chronic pain at the harvest site [43]. Allograft is usually readily available and avoids the morbidity associated with autograft harvest. Recent studies show comparable fusion rates with allograft and autograft [44, 45]. The disadvantages of using allograft include the risk of disease transmission and the increased cost. Fibular strut allografts can be used to reconstruct the defect following multilevel corpectomy. Synthetic bone graft substitutes are relatively new agents that have been used alone and in combination with autograft or allograft. When used alone synthetic graft substitutes avoid the complications of harvesting and disease transmission associated with autograft and allograft. One such agent, recombinant human bone morphogenetic protein-2 (rhBMP-2), has shown promising results in clinical trials [46]; however, there are still some concerns over its safety with a number of studies showing increased complications related to local and systemic inflammatory responses [47, 48] and reports of clinically significant neck swelling leading to acute airway compromise and dysphagia [49]. Synthetic bone graft substitutes are also relatively expensive.

## 6. Summary

Cervical spondylosis is a common problem encountered by the orthopaedic surgeon. Surgical decompression can be achieved through a multitude of procedures using either an anterior or posterior approach. The type of procedure carried out is dependent on a number of different variables including extent and location of pathology, previous surgery, congenital canal stenosis, and the presence of preoperative axial neck pain. Satisfactory surgical outcome will result in long-term amelioration of cervical radiculopathic and myelopathic symptoms with few postoperative complications.
